# Detour technique, Dipping technique, or IIeal bladder flap technique for surgical correction of uretero-ileal anastomotic stricture in orthotopic ileal neobladder

**DOI:** 10.1590/S1677-5538.IBJU.2013.0086

**Published:** 2015

**Authors:** Mohamed Wishahi, Hossam Elganzoury, Amr Elkhouly

**Affiliations:** 1 Department of Urology, Theodor Bilharz research Institute, Cairo, Egypt

**Keywords:** Urethral Stricture, Urinary Bladder, Surgical Flaps

## Abstract

**Background:**

Uretero-ileal anastomotic stricture (UIAS) is a urological complication after ileal neobladder, the initial management being endourological intervention. If this fails or stricture recurs, surgical intervention will be indicated.

**Design and Participants:**

From 1994 to 2013, 129 patients were treated for UIAS after unsuccessful endourological intervention. Unilateral UIAS was present in 101 patients, and bilateral in 28 patients; total procedures were 157. The previous ileal neobladder techniques were Hautmann neobladder, detubularized U shape, or spherical shape neobladder.

**Surgical procedures:**

Dipping technique was performed in 74 UIAS. Detour technique was done in 60 renal units. Ileal Bladder flap was indicated in 23 renal units. Each procedure ended with insertion of double J, abdominal drain, and indwelling catheter.

**Results:**

Follow-up was done for 12 to 36 months. Patency of the anastomosis was found in 91.7 % of cases. Thirteen patients (8.3%) underwent antegrade dilatation and insertion of double J.

**Conclusion:**

After endourological treatment for uretero-ileal anastomotic failure, basically three techniques may be indicated: dipping technique, detour technique, and ileal bladder flap. The indications are dependent on the length of the stenotic/dilated ureteral segment. Better results for long length of stenotic ureter are obtained with detour technique; for short length stenotic ureter dipping technique; when the stenotic segment is 5 cm or more with a short ureter, the ileal tube flap is indicated. The use of double J stent is mandatory in the majority of cases. Early intervention is the rule for protecting renal units from progressive loss of function.

## INTRODUCTION

Uretero-ileal anastomotic stricture (UIAS) in orthotopic ileal neobladder may develop in 4-10 % of cases in the follow-up period of 6-36 months (1–6). Various techniques were described to treat this complication which were mainly endourological, but not always successful (1, 4-9). The incidence of stricture would result in any of the described techniques of uretero-ileal anastomosis as Camey procedure, Hautman neobladder, Studer limb, chimney technique, extramural tunnel, and the direct refluxing techniques. Uretero-ileal anastomotic stricture is mostly caused by ischemia of the lower third of the divided ureter during the procedure of radical cystectomy, or due to improper surgical technique for anastomosis of the ureter to the constructed ileal neobladder. Uretero-ileal stricture is a challenging problem as it will lead to dilation of the affected renal unit, recurrent urinary tract infection (UTI), and ultimately loss of kidney function. The primary treatment is placement of a percutaneous nephrostomy tube followed by antegrade dilatation of the stricture and insertions of double J ureteral stent (4–8). The endourological procedure would fail due to impassable stricture, or the stenosis would recur soon after removal of the double J stent. The surgical approaches and standardization of the techniques for surgical correction of UIAS were not described before. Our goal is to describe our experience in the surgical management of uretero-ileal strictures.

## MATERIALS AND METHODS

From 1994 to 2013, we performed surgical correction of uretero-ileal stricture in 129 patients after radical cystectomy for invasive carcinoma of the bladder with orthotopic ileal neobladder by different techniques that included detubularized U-shaped neobladder, Hautman neobladder, or spherical shape neobladder. Most of these patients were referrals from different hospitals where the cystectomy procedure and the ileal neobladder were done by other urologists. Part of the materials and the surgical procedures were done in private hospitals to which the authors are affiliated to. Patients data were retrieved from hospital and private archives and extracted from doctoral theses that were completed before the year 2004. Patients included 112 men and 17 women. Total number of renal units affected by UIAS was 157. The incidence of bilateral stenosis was 28 out of 129 patients. Left side stricture was 68%, while the right side was 32%. The median interval to diagnosis of stricture was 8 months.

Investigations at presentation included laboratory work for renal function, liver function, prothrombin concentration, CBC, hepatitis markers, blood sugar, urine analysis and bacteriology. Imaging studies included abdominal ultrasonography and non-contrast CT-urography.

Initial intervention was ultrasound-guided placement of a percutaneous nephrostomy tube for drainage of the obstructed kidney, antibiotics being administered when urine was infected. Some cases referred to us had undergone insertion of nephrostomy tube and unsuccessful endourological procedure to insert ureteric stent.

Antegrade urography was done to evaluate the site and length of the stricture segment, urine cytology from the obstructed renal unit and the neobladder, cystoscopy was done to exclude cancer recurrence in the ileal neobladder pouch. Endourological management to treat the obstruction was tried first by passing a guide wire. After successfully passing the guide wire, the obstructed ureter was dilated by balloon catheter, followed by insertion of double J ureteral stent being left for 30 days. Development of restenosis, manifested by dilatation of the upper tract and/or recurrent UTI was an indication for surgical intervention.

**Figure 1:**
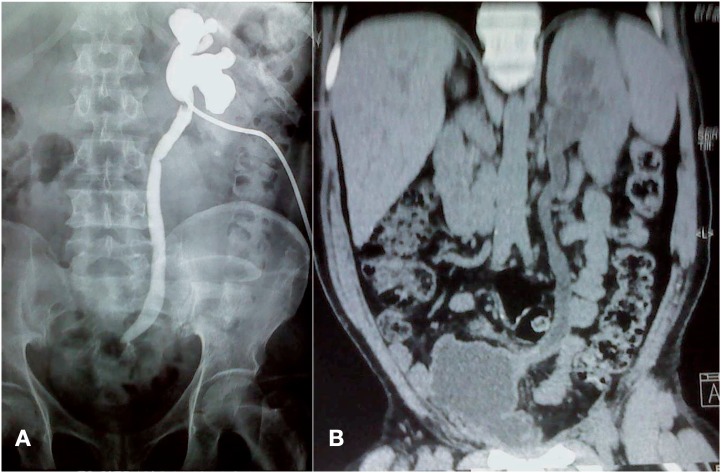
Uretero-ileal anastomotic stricture. (A) Antegrade urogram showing complete obstruction of the ureteroileal anastomotic stricture. (B) CT urography showing obstructed uretero-ileal anastomosis with dilatation of the renal unit.

**Figure 2:**
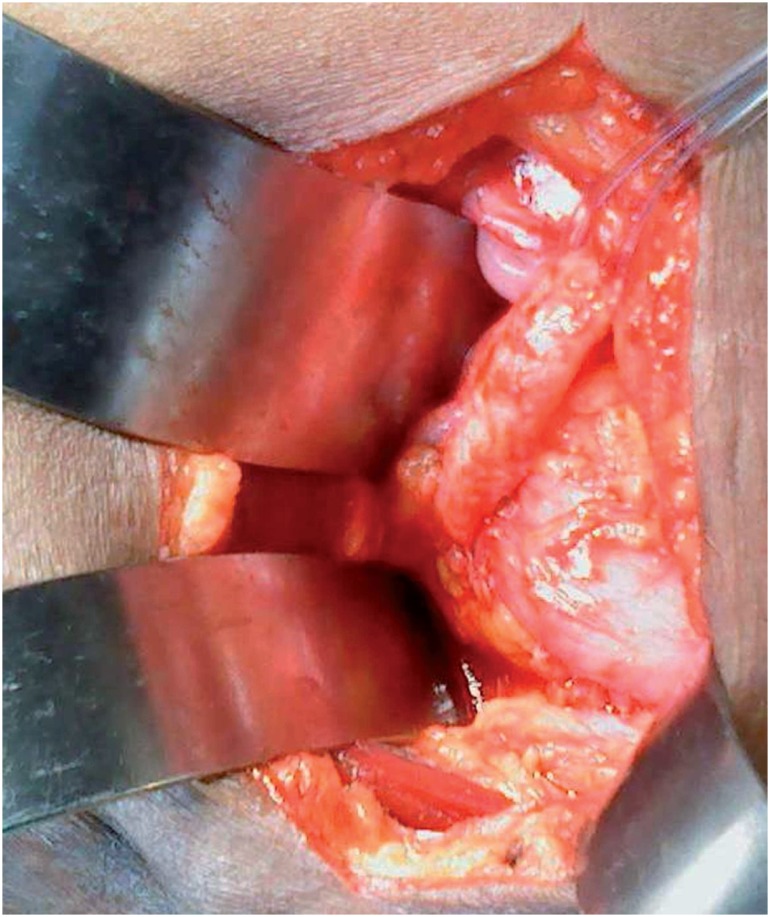
Intraoperative view showing the distended obstructed ureter and the distended neobladder as the ureter and the bladder were filled with saline solution via the nephrostomy tube and the bladder catheter.

Inclusion criteria in this series of 157 renal units were: 1) Complete obstruction of the ureter with no passage of the contrast media to the neobladder shown in antegrade urogram (Figure-1A); CT-urography confirming the complete ureteral obstruction and the length of the stenotic segment (Figure-1B); 2) Recurrent uretero-ileal anastomotic stricture following endourological dilatation and insertion of double J, with recurrent attacks of acute pyelonephritis.

### Surgical Technique

#### Incision and Identification of the Ureter and Neobladder

The operation is done under regional or general anesthesia, the patient being positioned in the supine position with tilting up the operating side for 15 degrees. The incision is 10 cm long on a line connecting the anterior superior iliac spine to the pubic tubercle; it is a muscle cutting incision or pararectal incision. The approach is trans-peritoneal, intestinal loops are displaced up in the abdominal cavity, the sigmoid colon in the left side is pushed laterally. The obstructed renal unit is infused with saline through the nephrostomy tube, the ureter will be seen in the operative field expanding and will be felt tense on palpation. Ureter will be circumferentially separated from adhesion and anchored with 6 Fr catheter. The ileal neobladder is filled with saline solution via the indwelling catheter to expose the uretero-ileal anastomotic site. The lower ureter is dissected from adhesion till it reaches the stenotic segment (Figure-2). The length of the stenotic segment is measured and according to the distance between the distal end of the dilated ureter and the neobladder the procedure is selected.

### Detour Technique

**Figure 3:**
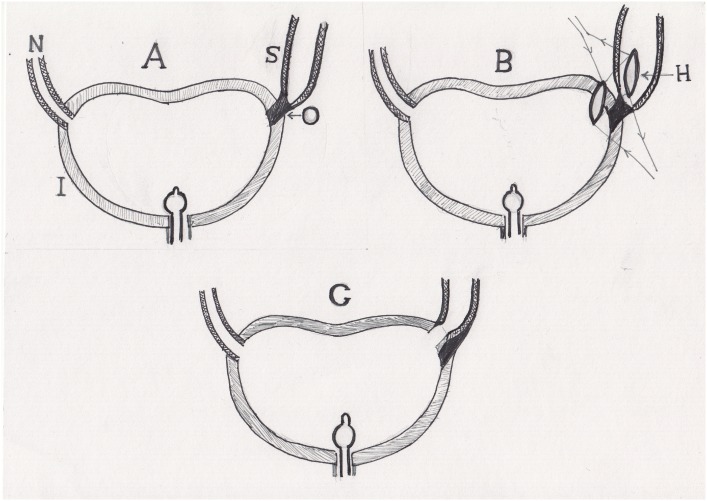
Detour technique. (A) The stenosed short ureter and the obliterated segment. (B) Two hiatus are created in the ureter and in the neobladder facing each other. (C) Two hiatus are connected with interrupted sutures in a side to side fashion.

Detour means side way, not the straight main street. The technique is illustrated in Figure-3. It was done in 60 uretero-ileal anastomotic strictures. The dilated ureter is followed down to the stenotic site. If the ureter has an insufficient length to reach the neobladder when it is empty and if cutting the ureter will not allow tension-free anastomosis, then the detour technique is performed. The site of the anastomosis is selected in the neobladder which is facing the lowest point in the dilated ureter. The ureter is not disconnected from the stenotic segment which will act as a fixation point. The width of the opening in the neobladder will be 1-2cm length. The mesenteric vessels of the mesentery of the ileal neobladder which is crossing the hiatus will be controlled by coagulation or multiple 4/0 interrupted sutures. A 1-2cm opening is made in the ureter facing the neobladder hiatus. Double J ureteric stent is passed to the kidney and the neobladder. The edges of the two hiatus are connected with interrupted 3 zero polyglycolic acid sutures. An abdominal drain is placed at the site of operation, and an indwelling urethral catheter is passed to the ileal neobladder.

### Dipping Technique

**Figure 4:**
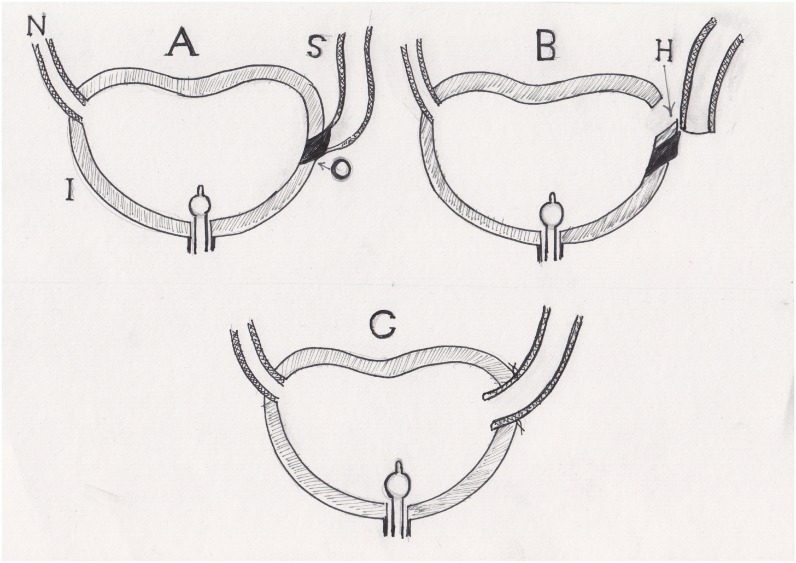
Dipping technique. (A) The stenosed long ureter and short obliterated segment. (B) The ureter is disconnected from the obliterated segment, and a hiatus is made in the ileal neobladder. (C) The ureter is implanted in the new bladder by dipping it in the hiatus; interrupted sutures are made.

**Figure 5:**
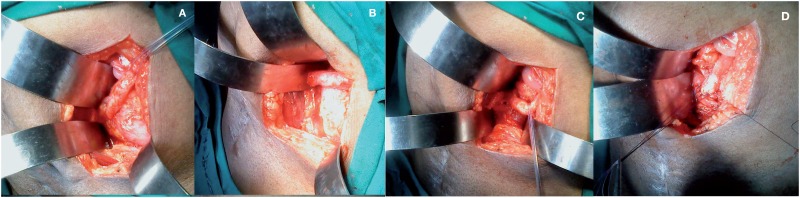
Intra-operative steps of dipping technique. (A) The obstructed ureter is identified and the site of obliterated segment is viewed. (B) The obstructed ureter is disconnected from the obliterated segment. (C) A hiatus is made in the neobladder to receive the dilated ureter. (D) The ureter is dipped into the neobladder, interrupted seromuscular sutures are made to fix the ureter to the ileal neobladder.

The technique is performed as illustrated in Figures 4 and 5. The dilated ureter is followed down to the stenotic site. When the ureter has sufficient length to reach the neobladder when it is empty, then the dipping technique is performed. A hiatus site in the neobladder is chosen being equal to the diameter of the dilated ureter; interrupted sutures are made around the hiatus to control the possible mesenteric vessels crossing this area. Double J ureteric stent is passed to the kidney and the neobladder; the ureter is dipped into the neobladder and fixed to it with 4-6 interrupted sutures with 3/0 polyglycolic acid sutures that are passed from the seromuscular layers of the ureter to the wall of the neobladder. An indwelling catheter is left in the neobladder, abdominal drain is placed in the operation site. The abdominal incision is closed in layers.

### Ileal Bladder Flap Technique

**Figure 6:**
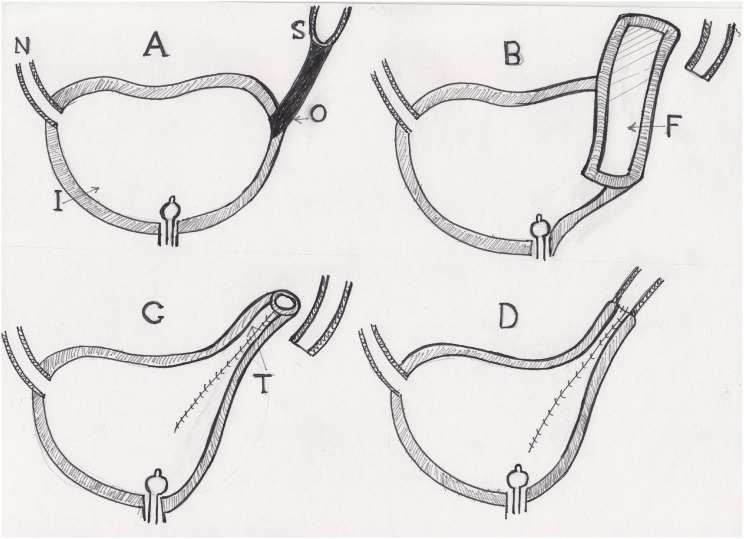
Ileal bladder flap technique. (A) Short ureter and long obliterated segment. (B) The ureter is disconnected from the stenosed segment. An ileal flap is created from the lateral side of the neobladder to avoid mesenteric vessels, its base is equal to the circumference of the dilated ureter. (C) The flap is fashioned to form a tube. (D) The newly created tube is anastomosed to the cut end of the dilated ureter.

This surgical technique was applied in 23 uretero-ileal strictures and was indicated by complete obliteration of the ureteric segment, lengthy stricture, and short dilated ureter. The ureter is followed down to the stenotic segment, the bladder is filled with saline via the indwelling catheter, and the length of the stenotic segment is measured, the range of 4-5cm or more being an indication for this technique. Details of the technique are illustrated in Figure-6. A quadrangular flap is selected from the lateral side of the ileal neobladder, the width of the free limb being equal to the circumference of the dilated ureter. Interrupted sutures are taken around the edges of the flap to control the possible mesenteric vessels crossing this area. A tube flap is fashioned using interrupted 3/0 sutures. An opening is made in the most distal part of the dilated ureter. The stenotic segment is not disconnected from the dilated ureter as it will act as a fixation band ensuring tension-free anastomosis. Double J ureteric stent is passed to the kidney and the neobladder; the ileal tube is anastomosed end to side to the dilated ureter with interrupted sutures. An indwelling catheter is left in the neobladder, and an abdominal drain is placed in the site of operation.

### Post-operative Care and Follow-up

In the three techniques, the indwelling urethral catheter was removed after one week post-operatively, if the abdominal drain was dry. If there was urine leakage, the indwelling catheter and the abdominal drain were left in place for another 5 days. The double J stent was removed after 30 days from removal of the indwelling catheter. Follow-up was done every 3 months with abdominal ultrasonography, every 6 months with CT-urography. Follow-up continued for 12-36 months with an average of 25 months.

## RESULTS

Surgical intervention for uretero-ileal stricture was done in 157 ureters. Three techniques were applied, the choice of either technique depending upon the length of the stenotic segment, and the length of the dilated ureter whether it would allow a tension free-anastomosis of the ureter to the neobladder. If so, the dipping technique was applied. In case the obstructed ureter was short and the stenotic segment was 2-3cm, the detour technique was applied. In presence of long stenotic segment of 5cm with short ureter, a tube flap technique was indicated. In patients with bilateral stricture, each side was dealt with independently.

Dipping technique was done in 74 renal units (47.1%), detour technique in 60 renal units (38.2%), and ileal tube flap ureteroplasty in 23 renal units (14.7%). The indwelling catheter and abdominal drain were removed after 7 days if there was no leakage. The double J ureteric stent was removed after 30 days of removal of the indwelling catheter. 10 patients were reported to have urine leakage following removal of the indwelling catheter. Re-insertion of the catheter was done for another 5 days where the leakage stopped. Postoperative follow-up for 12-36 months showed resolution of the pelvic-calyceal dilatation and no re-stricture in 91.7 % of cases. Thirteen patients (8.3%) showed unresolved dilatation of the upper tract and recurrent UTI with febrile episodes; they underwent antegrade ballon dilatation of the uretero-ileal region and insertion of double J for 3 months. In 8.3% of cases, no restenosis was recorded in the follow-up period.

## DISCUSSION

Uretero-ileal anastomotic stricture is a challenging urological complication after orthotopic ileal neobladder following radical cystectomy. Such complication will ultimately lead to loss of kidney function. In our series, we treated 157 renal units in 129 patients with failed endourological procedures. Schöndorf et al. (1), in their study on 74 patients with 85 obstructed renal units due to uretero-ileal anastomotic stricture, concluded that open surgical revision produces better results than primary invasive endourological intervention for uretero-ileal stricture. In our study the enrolling criteria was the failed endourological intervention or complete occlusion of the stricture segment. Stenzel et al. (2) reported that occurrence of uretero-ileal stricture depends on the technique used for construction of ileal neobladder; in their series they reported the incidence to be between 2.7 to 3.8%. In our series, most of the cases were referred from other hospitals university hospital as well as private sector. Our retrospective study covers the period between 1994 till 2013. Echo et al. (5) reported the incidence of uretero-ileal anastomotic stricture to be 5.2% and 18.3% in two groups of patients who underwent ileal neobladder and stenting of the uretero-ileal anastomosis with double J or open-end ureteric stent. They emphasised the frequency of occurrence of stricture. Maksiomovic et al. (6) had a 73.5 % success for endourological treatment of uretero-ileal anastomotic stricture and stated that the success of the endourological procedure was based upon morphology of the stricture. In our series, the completely obstructed stricture had failed endourological intervention. Echo et al. (5) in their series reported a failure rate of 26.5% which necessitated open surgical intervention. Liatsikos et al. (8) described the use of a self-expandable metal stent for uretero-ileal anastomotic stricture in 18 patients; pre-condition of feasibility of their technique being the primary patency of the stricture.

In our series, we applied three techniques for the open surgical treatment of uretero-ileal stricture; application of either technique was decided on the length of the stenotic segment and the length of the dilated ureter.

The present study is, to our knowledge, the largest series for the challenging complication of ureteroileal anastomotic stricture after orthotopic ileal neobladder. We described three different surgical techniques that covered the different possibilities of operative findings. Contrary to other authors (4), in our series we did not meet a condition that indicated to use an intestinal ileal segment or to perform complete ileal replacement of the ureter in order to bridge the obstructed segment. The three techniques fulfilled the criteria of direct access to the targeted site of stricture, avoided unnecessary dissection, and secured a tension-free anastomotic suture line.

## CONCLUSIONS

The three techniques described for treatment of uretero-ileal anastomotic stricture after radical cystectomy for bladder cancer and ileal neobladder replacement appear to be feasible surgical procedures. Dipping technique, detour technique and ileal bladder flap were applied to three potential morphological types of ureteric stenosis. Short and long term follow-up of the three procedures showed success and no recurrence of stenosis in 91.7% of cases. 8.3% renal units needed a second procedure of endourological dilatation and insertion of double J, in these cases there was no obliteration of the anastomotic site and not needed secondary operation. However, surgical treatment of uretero-ileal stricture should be the second step after unsuccessful endourological intervention, or recurrent stricture. An absolute indication to resort to surgical intervention is the radiological finding of complete obstruction of the stricture. Early intervention to treat uretero-ileal stricture will save renal units from progressive loss of function.

## ABBREVIATIONS

UIAS: = Ureter-ileal anastomotic stricture
UTI: = Urinary tract infection.
